# Reliabilität der Hornhauttomographie nach Implantation von intrakornealen Ringsegmenten bei Keratokonus

**DOI:** 10.1007/s00347-020-01074-w

**Published:** 2020-03-05

**Authors:** C. Matar, L. Daas, S. Suffo, A. Langenbucher, B. Seitz, T. Eppig

**Affiliations:** 1 Klinik für Augenheilkunde, Universitätsklinikum des Saarlandes UKS, Kirrberger Str. 100, 66421 Homburg/Saar, Deutschland; 2 Institut für Experimentelle Ophthalmologie, Universität des Saarlandes, 66421 Homburg/Saar, Deutschland; 3 AMIPLANT GmbH, Haidling 1, 91220 Schnaittach, Deutschland

**Keywords:** Keratokonus, INTACS, Pentacam, Vorderaugenabschnitts-OCT, Casia 2, Reproduzierbarkeit, Keratoconus, INTACS, Pentacam, Anterior Segment-OCT, Casia 2, Reproducibility

## Abstract

**Hintergrund und Ziel:**

Intrakorneale Ringsegmente (ICRS) sollen die Progression des Keratokonus (KK) aufhalten. Diese Aussage zur Progression erfordert jedoch Kenntnisse über die Reproduzierbarkeit der angewendeten Messverfahren. Ziel dieser Studie war es, die Reproduzierbarkeit tomographischer Parameter in Augen mit Keratokonus (KK) nach femtosekundenlasergestützter INTACS-Implantation (fs-INTACS) zwischen 2 Hornhauttomographen zu vergleichen.

**Patienten und Methoden:**

19 KK-Augen wurden eingeschlossen. 5 Messungen wurden mit dem Scheimpflug-Tomographen Pentacam HR und dem optischen Kohärenztomographen (VA-OCT) Casia 2 durchgeführt. Zielgrößen waren die Reproduzierbarkeit und die Vergleichbarkeit der Messungen von (1) keratometrischem Brechwert der Hornhautvorder- und (2) -rückfläche, (3) maximalem keratometrischem Brechwert, (4) zentraler Hornhautdicke und (5) Hornhautdicke an der dünnsten Stelle zwischen beiden Geräten.

**Ergebnisse:**

Die mittlere Differenz (Pentacam minus VA-OCT) von (1), (2), (3), (4) und (5) lag bei 0,67 dpt, 0,41 dpt, 3,4 dpt, 1,5 µm und 11,8 µm. Die mittlere SD der 5 Wiederholungen für (1), (2), (3), (4) und (5) lag bei 0,20 dpt/0,20 dpt, 0,10 dpt/0,07 dpt, 0,75 dpt/0,5 dpt, 6,5/2,4 µm (*p* = 0,007) und 7,3 µm/1,9 µm (*p* = 0,001) für Pentacam/Casia 2. Cronbach’s α war für alle Geräte und Parameter besser als 0,98.

**Schlussfolgerung:**

Casia 2 und Pentacam ermöglichen beide eine zuverlässige Beurteilung der Hornhautbrechkraft bei KK nach fs-INTACS-Implantation. Die Reproduzierbarkeit war nur für die Hornhautdickenmessung mit Casia 2 signifikant besser. Pentacam zeigte signifikant höhere Werte für die Brechkraft der Hornhautvorder- und Rückfläche und misst signifikant dicker an der dünnsten Stelle im Vergleich zu Casia 2.

## Abstract (en)

**Background and objective:**

Intracorneal ring segments (ICRS) are believed to stop the progression of keratoconus (KC). This statement on progression, however, requires knowledge about measurement reproducibility. The purpose of this study was to compare the reproducibility of tomographic parameters in eyes with KC after femtosecond laser-assisted implantation of INTACS (fs-INTACS) using two different devices and to determine which is more reliable for the follow-up of these patients.

**Patients and methods:**

In this study 19 KC eyes were included and repeatedly examined 5 times with the Scheimpflug topography Pentacam HR and the Casia 2 optical coherence tomography (VA-OCT) devices. Outcome measures included the reproducibility and comparability of measurements between the two devices of (1) keratometric refractive power of the anterior cornea and (2) posterior cornea, (3) maximum keratometric refractive power, (4) central corneal thickness and (5) corneal thickness at the thinnest site.

**Results:**

The mean differences (Pentacam minus Casia 2) of (1), (2), (3), (4) and (5) were 0.67 dpt, 0.41 dpt, 3.4 dpt, 1.5 µm and 11.8 µm, respectively. The mean SDs of the 5 repeat measurements for (1), (2), (3), (4) and (5) were 0.20 dpt/0.20 dpt, 0.10 dpt/0.07 dpt, 0.75 dpt/0.5 dpt, 6.5 µm/2.4 µm (*p* = 0.007) and 7.3 µm/1.9 µm (*p* = 0.001) for Pentacam and Casia 2, respectively. Cronbach’s alpha was better than 0.98 for both devices and all parameters.

**Conclusion:**

Both Casia 2 and Pentacam enable a reliable assessment of the corneal refractive power in KC after fs-INTACS implantation; however, the reproducibility was significantly better with Casia 2 only for the measurement of corneal thickness. The Pentacam showed significantly higher values for the mean anterior and posterior corneal refractive power and measured significantly thicker at the thinnest point of the cornea compared to Casia 2.

Um die Progression des Keratokonus aufzuhalten, werden unter anderem intracorneale Ringsegmente (ICRS) eingesetzt. Um jedoch eine Aussage zur Keratokonusprogression treffen zu können, sind Kenntnisse über die Reproduzierbarkeit der Messverfahren unbedingt erforderlich. Ziel dieser Arbeit war es, die Reproduzierbarkeit tomographischer Parameter in Augen mit KK nach femtosekundenlasergestützter INTACS-Implantation, vergleichend für 2 verschiedene Hornhauttomographiegeräte zu beurteilen.

## Hintergrund und Fragestellung

Unter dem Begriff Keratokonus (KK) wird ein Spektrum kornealer Veränderungen zusammengefasst, welches als gemeinsames Kennzeichen eine kegelförmige Wölbungsanomalie der Hornhaut aufweist. Die Hornhauterkrankung ist in der Regel progressiv und manifestiert sich meist zwischen dem 15. und 30. Lebensjahr. Sie führt zu einer Verdünnung des Hornhautstromas und im Endstadium zu Narbenbildungen der zentralen Kornea und damit zu einer erheblichen Abnahme der Sehschärfe und Lebensqualität [15]. Der Keratokonus ist eine in der Regel bilateral auftretende Erkrankung mit einer hohen Wahrscheinlichkeit einer Seitenasymmetrie zwischen beiden Augen [9]. Obwohl schon seit langer Zeit als nichtinflammatorische Erkrankung identifiziert, wird in den letzten Jahren immer mehr belegt, dass der Keratokonus teilweise eine inflammatorische Ätiologie hat [11].

**Figure 1:**
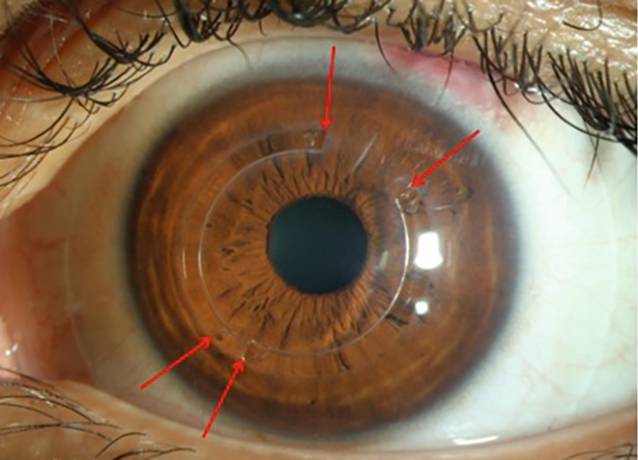

INTACS (Addition Technology Inc., Fremont, CA, USA), eine Art intrakornealer Ringsegmente (ICRS), bestehen aus bogenförmigen elliptischen Segmenten aus Polymethylmethacrylat (PMMA), die chirurgisch in das tiefe Hornhautstroma (80 % Tiefe) des Auges eingeführt werden, um die Hornhaut mechanisch zu stabilisieren und die zentrale Hornhaut abzuflachen ([2]; Abb. 1). Ursprünglich für die refraktive Korrektur von leichter Myopie konzipiert, gelten sie heute als fester Bestandteil der stadiengerechten Therapie des Keratokonus (KK).

Bereits 2001 berichteten Colin et al. zum ersten Mal über die Sicherheit und Wirksamkeit von INTACS nach Implantation bei 10 Keratokonuspatienten [5]. Seitdem hat dieses Verfahren an Beliebtheit bei refraktiven Chirurgen gewonnen. Für Keratokonuspatienten mit Kontaktlinsenintoleranz stellen INTACS die Alternative zu einer frühen Keratoplastik dar [7]. Weitere Indikationen sind Post-LASIK-Keratektasie und die pellucide marginale Degeneration [8]. Voraussetzungen für die Implantation sind das Fehlen von zentralen Hornhautnarben, eine Obergrenze des steilsten K‑Wertes von nicht mehr als 58 dpt und eine minimale Hornhautdicke von 400 μm an der Implantationsstelle. Da die Ausdünnung bei der pelluciden marginalen Degeneration limbusnah liegt, ist die INTACS-Implantation mit dem Femtosekundenlaser aufgrund der Applanation bzw. des gekrümmten Interfaces deutlich erschwert.

Es ist bekannt, dass INTACS sowohl die unkorrigierte und die bestkorrigierte Sehschärfe verbessern und den Astigmatismus verringern sollen [8, 13]. Zum anderen scheinen sie auch die Progression des KK zu hemmen. Bedi et al. publizierten 2012 refraktive und topographische postoperative Ergebnisse 5 Jahre nach konsekutiver fs-INTACS-Implantation bei 105 Augen von KK-Patienten [1]. Ferner wurde zwischen Augen mit dokumentierter präoperativer Progression (Zunahme im steilen Keratometriewert [Ks] um mehr als 1,00 dpt über 12 Monate präoperativ) und Augen ohne dokumentierte präoperative Progression unterschieden. Dabei zeigte sich insgesamt bei 91,3 % der Augen beider Gruppen und bei 92,9 % der Augen mit dokumentierter präoperativer Progression ein stabiler postoperativer Verlauf zwischen 1 und 5 Jahren postoperativ. Eine postoperative Progression wurde laut Autoren als Zunahme der Ks um mehr als 1 dpt über einen Zeitraum von 4 Jahren (zwischen 1 und 5 Jahren postoperativ) definiert. Zwar bislang noch nicht einheitlich definiert, spricht man laut Beschluss des gemeinsamen Bundesausschusses, die im Januar 2019 in Kraft trat, von einer Progression, wenn innerhalb von 12 Monaten eine Zunahme der maximalen Hornhautbrechkraft (K_max_) um mehr als 1 dpt oder eine Zunahme des durch die subjektive Refraktion bestimmten Astigmatismus um ≥1 dpt oder eine Abnahme der Basiskurve der bestsitzenden Kontaktlinse um ≥0,1 mm nachgewiesen werden kann [12]. In der Literatur wurden zahlreiche potenzielle Hornhautparameter zur Identifikation einer Progression beschrieben [3, 6]. *Um eine Aussage zur KK-Progression zu treffen, sind jedoch Kenntnisse über die Reproduzierbarkeit der Messverfahren unbedingt erforderlich. *Unter Reproduzierbarkeit wird die Übereinstimmung von Messergebnissen verstanden, die unter (leicht) veränderten Bedingungen mit der gleichen Methode gemessen werden. Meist sind dies andere Umgebungsbedingungen, andere Positionierung des Patienten oder andere Untersucher.

Das Ziel dieser Arbeit war es, die Reproduzierbarkeit tomographischer Parameter in Augen mit KK, die sich einer femtosekundenlasergestützten INTACS-Implantation (fs-INTACS) untergezogen hatten, vergleichend für 2 verschiedene Hornhauttomographiegeräte zu beurteilen.

## Studiendesign und Untersuchungsmethoden

Insgesamt wurden 19 Augen mit der Diagnose Keratokonus von 12 Patienten (Durchschnittsalter 33 ± 10 Jahre [20 bis 55 Jahre]) eingeschlossen. Alle Patienten hatten eine fs-INTACS-Implantation zwischen 2014 und 2018 durch 2 erfahrene Operateure aufgrund von KK von Stadium 2 oder größer (gemäß topographischer Keratokonusklassifikation [TKC]) erhalten. Bei allen Patienten wurde präoperativ die Hornhautdicke an der Implantationsstelle (6-mm-Zone) mit dem VA-OCT gemessen und der Tunnel mit dem Femtosekundenlaser kreiert, um die INTACS-Implantate in der optischen 6‑ bis 7‑mm-Zone zu implantieren. Die Ringsegmente wurden immer tief im kornealen Stroma (80 % von der dünnsten Stelle der Hornhaut in der 6‑mm-Zone) implantiert. Es wurden folgende Einstellungen am Femtosekundenlaser vorgenommen: Tiefe des Tunnels 80 % der dünnsten Stelle der Hornhaut in der 6‑mm-Zone, Innendurchmesser 6,0 mm, Außendurchmesser 7,0 mm, Laserenergie 1,5 mJ. Die Dicke der Ringsegmente wurde entsprechend einem Nomogramm des Herstellers auf die individuelle Hornhaut abgestimmt. Bei allen Patienten wurde postoperativ eine Verbandskontaktlinse (AIR OPTIX® Night&Day Aqua, Ciba Vision GmbH, Großwallstadt, Deutschland) 1 Woche lang aufgesetzt. Dazu wurden Prednisolon (Inflanefran forte® 10 mg/ml, Allergan Pharmaceuticals Ltd., Irland) und Moxifloxacin Hydrochlorid 0,5 % (Vigamox, Alcon Pharma GmbH, Freiburg/Breisgau, Deutschland) 6‑mal täglich im Wechsel appliziert und die kortisonhaltigen Augentropfen anschließend stufenweise wöchentlich um 1 Tropfen reduziert. Konservierungsmittelfreie Befeuchtungstropfen (Optive UD Augentropfen; Allergan Pharmaceuticals) wurden zusätzlich bei allen Patienten initial 6‑mal täglich angesetzt. Es wurden jeweils 5 Wiederholungsmessungen mit dem Scheimpflug-Tomographen Pentacam HR (Oculus Optikgeräte GmbH, Wetzlar, Deutschland) und dem optischen Kohärenztomographen des vorderen Augenabschnitts (VA-OCT) Casia 2 (Tomey Corp., Nagoya, Japan) durchgeführt.

Alle Messungen wurden am gleichen Tag mit einer Pause von *5* *min* zwischen den einzelnen Messungen unter mesopischen Bedingungen, bei spielender Pupille und durch denselben Untersucher durchgeführt. Vorab wurden die Patienten ausführlich über die korrekte Positionierung des Kopfes auf die Kinnstütze sowie über den geraden Augenblick und Fixierung des Zielobjekts im Zentrum der Aufnahmekamera während der einzelnen Messungen aufgeklärt. Die Untersuchungen wurden gemäß den ethischen Grundsätzen der Deklaration von Helsinki durchgeführt.

Primäre Zielgrößen waren die Reproduzierbarkeit von *(1) mittlerem keratometrischem Brechwert der Hornhautvorderfläche (Km), (2) mittlerem Brechwert der Hornhautrückfläche (PKm), (3) maximalem keratometrischem Brechwert (K*_*max*_*), (4) zentraler Hornhautdicke (CCT) und (5) Hornhautdicke an der dünnsten Stelle (TCT)*.

### Statistische Analyse

Die erhobenen Werte wurden mittels SPSS® Version 24.0 (IBM, Armonk, USA) statistisch ausgewertet. Die Reproduzierbarkeit wurde als mittlere Standardabweichung (SD) zwischen den 5 Wiederholungsmessungen ausgedrückt. Der Unterschied in der Reproduzierbarkeit wurde mit einem paarigen Wilcoxon-Rangsummentest untersucht. *p*-Werte <0,05 wurden dabei als statistisch signifikant gewertet. Eine A‑priori-Fallzahlanalyse mit der Software G*Power (Erdfelder, Faul, & Buchner, HHU Düsseldorf, Deutschland) [10] ergab, dass für den Nachweis einer Effektgröße dz = 1 (entsprechend 0,25 dpt Unterschied in der Keratometrie) und einer geforderten Power von 95 % eine Fallzahl von 16 Fällen notwendig ist. Als Maß für die Reliabilität wurde zudem Cronbach’s α berechnet. Die Vergleichbarkeit beider Geräte wurde anhand Bland-Altmann-Plots dargestellt.

## Ergebnisse

Die Anzahl der Augen in den TKC-Gruppen 2|3|4 betrug 4|8|7. Bei 4 Augen wurde 1 Ringsegment und bei 15 Augen wurden 2 Ringsegmente implantiert. Der zeitliche Abstand zwischen der fs-INTACS-Implantation und der Messung betrug im Mittel 2,3 Jahre. Die Dicke des dickeren Ringsegments lag zwischen 300 und 450 μm.

Die mittleren Messwerte aus den 5 Wiederholungsmessungen der einzelnen Augen und Geräte sowie die dazugehörige Standardabweichung sind in Tab. 1 aufgeführt.

**Table 1:** 

ID	Casia 2					Pentacam HR				
	Km	PKm	CCT	TCT	K_MAX_	Km	PKm	CCT	TCT	K_MAX_
1	46,09 ± 0,18	−8,19 ± 0,04	501 ± 2	465 ± 2	52,02 ± 0,73	46,48 ± 0,08	−7,30 ± 0,10	493 ± 30	476 ± 32	57,78 ± 0,35
2	47,25 ± 0,14	−8,47 ± 0,06	481 ± 2	433 ± 1	53,15 ± 0,74	49,04 ± 0,21	−7,76 ± 0,05	483 ± 4	461 ± 5	60,94 ± 1,97
3	45,70 ± 0,17	−7,56 ± 0,06	457 ± 2	449 ± 1	50,88 ± 0,47	46,62 ± 0,08	−7,54 ± 0,11	473 ± 3	469 ± 4	55,24 ± 0,31
4	50,64 ± 0,13	−8,51 ± 0,02	433 ± 1	420 ± 1	56,77 ± 0,24	53,28 ± 0,08	−8,66 ± 0,05	439 ± 5	425 ± 5	62,34 ± 0,43
5	43,93 ± 0,14	−7,18 ± 0,04	527 ± 2	487 ± 4	49,30 ± 0,15	44,06 ± 0,17	−6,68 ± 0,39	509 ± 21	478 ± 28	54,42 ± 0,95
6	47,68 ± 0,27	−8,11 ± 0,04	437 ± 4	417 ± 3	53,86 ± 0,78	48,38 ± 0,08	−7,44 ± 0,05	442 ± 9	430 ± 8	56,08 ± 1,01
7	50,86 ± 0,19	−8,25 ± 0,06	451 ± 5	413 ± 5	59,22 ± 1,19	51,14 ± 0,26	−7,46 ± 0,05	445 ± 5	429 ± 5	63,04 ± 1,09
8	42,56 ± 0,11	−6,52 ± 0,05	471 ± 2	461 ± 2	49,16 ± 0,20	42,64 ± 0,05	−6,20 ± 0,10	488 ± 5	469 ± 13	48,70 ± 1,19
9	44,34 ± 0,05	−7,14 ± 0,10	509 ± 1	474 ± 2	49,29 ± 0,34	43,90 ± 0,20	−6,58 ± 0,04	514 ± 4	498 ± 3	49,36 ± 0,61
10	52,18 ± 0,16	−8,99 ± 0,05	424 ± 2	392 ± 2	66,35 ± 0,63	54,96 ± 0,21	−8,34 ± 0,05	433 ± 4	411 ± 2	74,56 ± 0,46
11	47,86 ± 0,27	−7,77 ± 0,08	457 ± 2	409 ± 0	59,11 ± 0,46	47,16 ± 0,55	−6,72 ± 0,16	457 ± 6	431 ± 1	63,84 ± 1,51
14	48,98 ± 0,17	−7,63 ± 0,03	474 ± 1	442 ± 0	58,92 ± 0,39	48,34 ± 0,21	−6,88 ± 0,04	471 ± 2	440 ± 5	63,66 ± 0,36
15	51,14 ± 0,25	−8,67 ± 0,08	453 ± 1	417 ± 1	65,00 ± 0,27	51,54 ± 0,67	−8,36 ± 0,11	439 ± 7	419 ± 8	62,36 ± 1,13
16	50,28 ± 0,09	−8,78 ± 0,03	333 ± 1	331 ± 1	60,18 ± 0,13	50,72 ± 0,20	−9,02 ± 0,04	329 ± 4	322 ± 4	59,98 ± 0,66
17	39,81 ± 0,57	−7,13 ± 0,26	478 ± 5	453 ± 2	46,81 ± 0,54	40,54 ± 0,15	−6,80 ± 0,14	479 ± 3	470 ± 2	51,04 ± 0,39
18	42,61 ± 0,57	−7,74 ± 0,26	451 ± 9	433 ± 6	54,98 ± 1,05	44,30 ± 0,19	−7,78 ± 0,16	456 ± 4	447 ± 4	57,76 ± 0,43
19	47,01 ± 0,08	−7,74 ± 0,06	460 ± 3	430 ± 1	51,55 ± 0,35	46,80 ± 0,14	7,50 ± 0,12	469 ± 2	455 ± 2	53,62 ± 0,34

*Km* mittlerer keratometrischer Brechwert der Hornhaut, *PKm* mittlerer Brechwert der Hornhautrückfläche, *CCT* Hornhautdicke am Apex, *TCT* Hornhautdicke an der dünnsten Stelle, *K*_*max*_ keratometrischer Brechwert der Hornhaut an der steilsten Stelle

**Figure 2:**
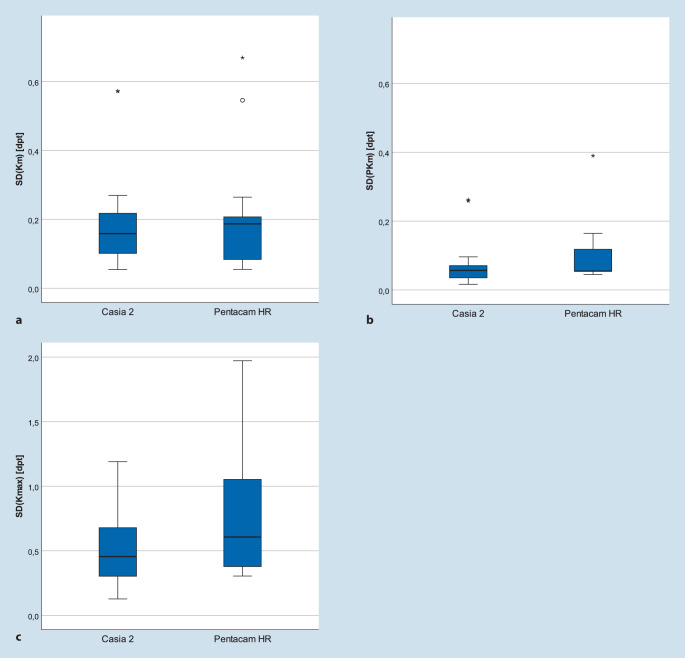

**Figure 3:**
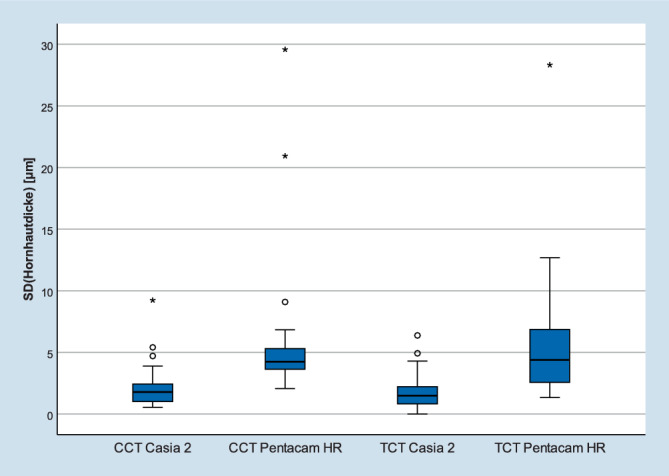

Die mittlere Standardabweichung (SD) des durchschnittlichen keratometrischen Hornhautbrechwerts (Km) betrug bei der Pentacam 0,20 und beim Casia 2 0,20 dpt (*p* = 0,629) (Abb. 2a). Der posteriore Hornhautbrechwert (PKm) zeigte eine mittlere SD von 0,10 vs. 0,07 dpt (*p* = 0,117) im Vergleich von Pentacam zu Casia 2 (Abb. 2b). Die mittlere SD des maximalen keratometrischen Hornhautbrechwerts (K_max_) lag bei 0,75 dpt bzw. 0,50 dpt für Pentacam und Casia 2 (*p* = 0,099). Die zentrale (CCT) und die dünnste Hornhautstelle (TCT) zeigten in der Pentacam im Vergleich zu Casia 2 eine mittlere SD von 6,5 vs. 2,4 µm (*p* = 0,007) und 7,3 vs. 1,9 μm (*p* = 0,001) (Abb. 3). Cronbach’s α war für beide Geräte und Parameter besser als 0,98.

**Figure 4:**
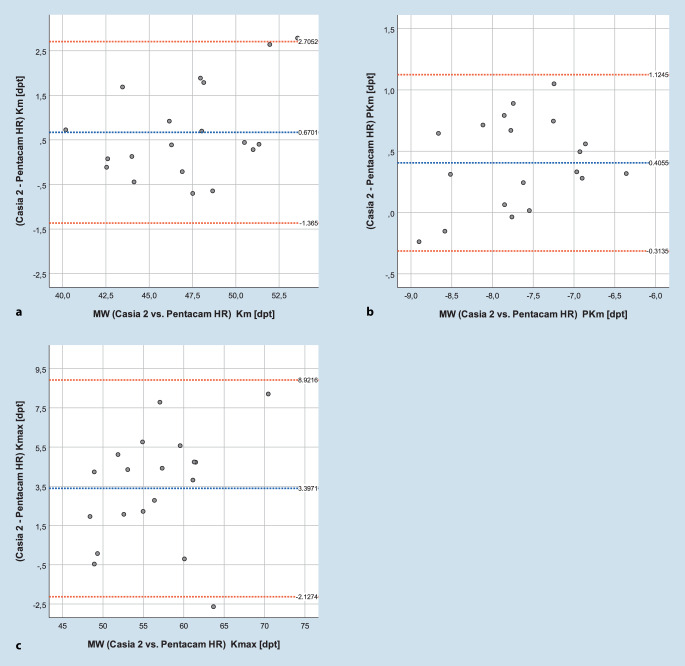

**Figure 5:**
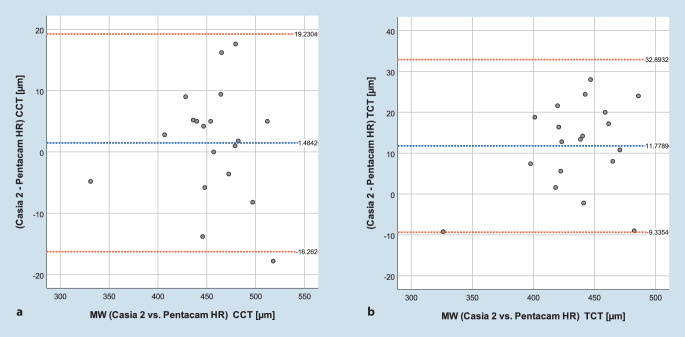

Die mittlere Differenz des anterioren bzw. posterioren mittleren Hornhautbrechwerts sowie des maximalen Keratometriewerts K_max_ zwischen beiden Geräten (Pentacam und Casia 2) lag bei 0,67 ± 1,03 dpt (*p* = 0,02), 0,41 ± 0,37 dpt (*p* = 0,001) und 3,40 ± 2,82 dpt (*p* = 0,01). Die mittlere Differenz der CCT und TCT lag bei 1,5 ± 9 µm (*p* = 0,396) und 11,8 ± 10,8 µm. Die Hornhautdicke an der dünnsten Stelle (TCT) wurde mit dem VA-OCT signifikant dünner gemessen als mit der Pentacam (*p* = 0,01). Die Grenzen der Übereinstimmung (Bland-Altmann) für die Hornhautbrechwerte sind in Abb. 4a–c, die für zentrale und dünnste Hornhautdicke in Abb. 5a, b dargestellt.

## Diskussion

Die Erkennung von Veränderungen eines Zustands mithilfe von Messmethoden kann nur dann gelingen, wenn die Messung hinreichend genau und reproduzierbar ist. Daher kann eine Progression eines Keratokonus nur dann sicher festgestellt werden, wenn die verwendeten Messmethoden eine höhere Reproduzierbarkeit aufweisen als der Grenzwert, der für eine Progression spricht.

Der Begriff der Reproduzierbarkeit („reproducibility“) wird oft mit dem Begriff der Wiederholbarkeit („repeatability“) gleichgesetzt, jedoch unterscheiden diese sich in einem wesentlichen Punkt: Die Reproduzierbarkeit kann nur unter leicht geänderten Bedingungen ermittelt werden [14]. Dies kann z. B. eine andere Positionierung des Patienten bedeuten, ein anderes Messgerät des gleichen Typs oder ein anderer Untersucher. In unserer Studie wurde zwischen den einzelnen Messungen jeweils die Position des Patienten in der Kinnstütze verändert. Zudem wurde eine Zeitspanne zwischen den 5 Untersuchungen eingehalten, die mindestens das 5‑Fache der Untersuchungsdauer beträgt (1 min Untersuchung, dann 5 min Pause, danach 1 min Untersuchung etc.).

Unsere Ergebnisse zeigen, dass beide Messgeräte bezüglich der mittleren Hornhautbrechwerte bei INTACS-Patienten eine hohe Reproduzierbarkeit von etwa 0,2 dpt aufweisen. Chen und Lam untersuchten die Reproduzierbarkeit von Pentacam-Messungen an Normalaugen zu 2 verschiedenen Zeitpunkten [4]. Vergleicht man diesen Wert mit dem „coefficient of repeatability“ (1,96-fache Standardabweichung) aus der Studie von Chen und Lam [4], so erreichen beide Geräte bei Augen nach INTACS-Implantation eine etwa halbierte Reproduzierbarkeit im Vergleich zu Normalaugen (±0,39 dpt im Vergleich zu ±0,21 dpt für Km). Hierdurch könnten wiederholt nachweisbare Veränderungen ≥0,5 dpt als Progressionsverdacht gewertet werden. Allerdings gilt zu beachten, dass Kontaktlinsen und diverse Medikamente Veränderungen an der Hornhaut bewirken können, die eine Progression vortäuschen können. Veränderungen von ≥1 dpt, wie in den aktuellen Leitlinien empfohlen, sind jedoch bei wiederholter Messung als Progression definierbar. Der maximale Hornhautbrechwert K_max_ zeigt eine deutlich größere Unsicherheit von 0,5 dpt beim VA-OCT und 0,75 dpt bei der Pentacam. Dieser Parameter wird üblicherweise zur Definition einer Progression herangezogen, wobei eine Veränderung von >1 dpt als Progression gewertet wird [12]. Bezieht man sich wieder auf den „coefficient of repeatability“ nach Chen und Lam [4], so wäre mit VA-OCT der Grenzwert zur Feststellung der Progression von 1 dpt anwendbar, im Falle der Pentacam müsste der Grenzwert jedoch auf bis zu 1,5 dpt angehoben werden. In jedem Fall empfiehlt es sich, die Aussage durch Wiederholung der Messung zu bestätigen [4]. Ebenfalls sehr deutlich fällt der Unterschied zwischen beiden Geräten bezüglich K_max_ mit rund 3,4 dpt aus, der vermutlich durch die unterschiedlichen Messverfahren und ggf. fehlerhafte Erkennung der Hornhautfläche (z. B. durch erhöhte Streuung in der Umgebung des Implantats bei der Pentacam) begründet ist.

Bezüglich der Hornhautdicke zeigt sich ein differenzierteres Bild, das VA-OCT weist hier eine höhere Reproduzierbarkeit nur bezüglich der Messung der Hornhautdicke auf. Die Bland-Altmann-Analyse zur Übereinstimmung zwischen beiden Geräten verdeutlicht, dass die Unterschiede zwischen beiden Geräten vergleichsweise groß ausfallen (mehrere Dioptrien bezüglich der Hornhautbrechung bzw. bis zu 20 µm bezüglich der Hornhautdicke). Die Pentacam zeigte signifikant höhere Hornhautbrechwerte als das Casia 2. Zudem misst die Pentacam die Hornhautdicke an der dünnsten Stelle signifikant dicker. Demnach können beide Geräte nicht als gegeneinander austauschbar betrachtet werden. Eine analoge Auswertung unserer Arbeitsgruppe an Keratokonusaugen ohne ICRS zeigte, dass die Standardabweichung bei wiederholter Messung mit zunehmendem TKC-Stadium zunimmt [17]. Außerdem wurde die Dicke mittels VA-OCT um 10–30 µm stadienabhängig dünner gemessen [17]. Eine Arbeit von Wonneberger et al. kam zu dem Schluss, dass die Streuung des K_max_-Wertes bei zunehmendem Keratokonusschweregrad ebenfalls zunimmt [18].

Schröder et al. untersuchten Pentacam HR und Casia SS-1000 in einer Serie von gesunden Augen in Bezug auf die kornealen Höhendaten und die Pachymetrie [16]. Sie stellten fest, dass die Pentacam HR hier die größte Reproduzierbarkeit aufwies, gefolgt vom Casia SS-1000. Die Auswertung bezog sich jedoch nicht auf die keratometrischen Brechwerte und ist daher schwer mit unserer Studie vergleichbar. In Übereinstimmung mit unserer Studie attestierten sie dem Casia SS-1000 eine höhere Reproduzierbarkeit bezüglich der Hornhautdickenmessung als der Pentacam HR.

Obwohl die Ultraschallpachymetrie immer noch als Goldstandard für die Hornhautdickenmessung gilt, handelt es sich um eine Kontaktmethode die mit einem erhöhten Risiko für Hornhautverletzungen und Infektionen insbesondere bei ektatischen Hornhäuten einhergeht. Inzwischen gilt die optische Messung der Hornhautdicke mehr und mehr als Standard. Diese erlaubt nicht nur, die Diagnose Keratokonus zu stellen bzw. die Kontraindikationen für die einzelnen therapeutischen Möglichkeiten zu erfassen (v. A. Crosslinking oder INTACS-Implantation), sondern auch den Verlauf präzise zu dokumentieren, um ggf. eine Eskalierung in der Therapie im Fall einer Progression zu rechtfertigen. Mehrere Studien verglichen die Hornhautdicke bei Augen mit Keratokonus mit verschiedenen Messgeräten. Bis jetzt ist uns jedoch keine Studie bekannt, die die Reproduzierbarkeit und Vergleichbarkeit der Hornhautdickenmessung und Keratometrie bei Keratokonusaugen mit INTACS-Implantaten zwischen 2 Tomographen untersuchte.

## Schlussfolgerung

Wir schlussfolgern daraus, dass zwar das VA-OCT Casia 2 und die Pentacam aufgrund der gegebenen Reproduzierbarkeit der Messwerte für die Verlaufsbetrachtung nach INTACS-Implantation geeignet sind, die Messwerte jedoch nicht gegeneinander ausgetauscht werden dürfen. Für eine Progressionsbetrachtung sollte daher immer das gleiche Gerät verwendet werden.
